# An In-Store Experiment on the Effect of Accessibility on Sales of Wholegrain and White Bread in Supermarkets

**DOI:** 10.1371/journal.pone.0151915

**Published:** 2016-03-24

**Authors:** René A. de Wijk, Anna J. Maaskant, Ilse A. Polet, Nancy T. E. Holthuysen, Ellen van Kleef, Monique H. Vingerhoeds

**Affiliations:** 1 Wageningen UR-Food & Biobased Research, Wageningen, The Netherlands; 2 Wageningen University, Marketing and Consumer Behaviour Group, Wageningen, the Netherlands; University of Cambridge, UNITED KINGDOM

## Abstract

Even though whole grain foods have various health benefits, consumers have been found not to eat enough of them. Nudging interventions are built on the premise that food purchases and consumption are strongly influenced by the environment in which decisions are made. Little research has been conducted to examine the influence of a small and inexpensive nudging intervention on bread choices in a real-life supermarket context. An in-store experiment was conducted in two six-week periods in two supermarkets to investigate the effects of accessibility on consumers’ purchase of healthier whole grain and other types of bread. In the high accessibility condition, healthier bread was placed in a more convenient location for the shopper on the left side of the shelves where it was encountered first. In the low accessibility condition, it was placed on the right side. There were consistent significant differences in sales between supermarkets, types of bread, day of the week, but not between low and high accessibility. Additional research is needed to better understand the effects of convenience and accessibility on bread choices.

## Introduction

The health benefits of whole grain foods are well documented [1–4]. Nevertheless, in many countries whole grain consumption is low compared to international recommendations [5–6]. Whole grain consumption has been promoted in various ways, such as by emphasizing or improving its affordability, healthiness, availability, taste and appearance [7–10]. In the last decade, there has been a considerable increase in interest in using insights from behavioural economics and psychology to change behaviour in a healthier direction, because a lot of behaviour is driven by habits and automatic routines. Every day, people are faced with numerous instances where they have to make food-related decisions such as whether they want to eat, what they want to eat, what the rest of the family want to eat, whether the food is healthy and safe, and whether the food is in line with their health goals (Wansink and Sobal [11] mention up to 200 food-related decisions that the average person has to take per day). Giving each of these questions sufficient thought is mentally exhausting, and there is a great temptation to fall back on automated and less healthy behaviours [12]. Moreover, most supermarket purchases are unplanned, i.e., they are based on in-store decisions. Estimates of unplanned purchases vary between studies. For example, Inman et al. estimate that 60.9% of purchases are unplanned [13].

Increasingly, interventions focus on arranging the decision-making contexts in such a way that they engage with consumers’ automatic and impulse-driven decision-making system. These so-called nudging interventions adapt the environment in which people make choices to help them make better choices, without appealing to people’s reason or forcing certain choices upon them [14–16]. The evidence base for nudging as a means to improve the healthiness of food choices is growing. Depending on the specific studies, increases or decreases in sales as a result of nudging may be as large as 28% [17]. So far, nudging studies have targeted especially homes, schools, grocery stores, and restaurants. The essence of the nudging approach is to change environments in such a way that the healthy choice becomes a more convenient, attractive, or normal choice [18]. This paper focuses especially on the convenience component of a nudge approach by adapting the shelf location of healthier and less healthy breads. Locating healthy foods at a prominent or eye-level place and the unhealthy options further away is one of the most frequently mentioned examples when nudges are defined and illustrated (see for example [14, 19–20]).

Convenience is the result of the ease, time, and comfort involved in obtaining a food and can be achieved in many ways. Small convenience changes in the environment, such as changing the distance to healthy or unhealthy foods, placing specific foods near the checkout, or changing the order of food in buffet lines, are typically not consciously noticed by consumers but have demonstrated effects on food choices and can assist in making the healthy choice the convenient choice [17, 21–22]. Research on the way shelves are stocked in grocery stores shows that a product’s absolute and relative shelf position strongly influences the choices that consumers make [23]. The more shelf space a type of product occupies or the more prominent the shelf location (e.g. at eye level or end of aisle display), the more items are sold [24].

The present study examines the convenience nudge principle in supermarkets to select healthier whole grain bread. The habit strength of bread purchases is typically high, as people have a repeated pattern of purchase [25]. Although nudging studies in canteen settings have shown effects of more prominent location of healthier foods [26–27], to our knowledge, no study has specifically attempted to nudge consumers’ bread purchases in a real-world supermarket setting. The current research extends prior research by exploring consumer purchase responses to a convenience nudge to encourage whole grain bread purchases in two supermarkets over an extended period of time. Healthier bread choices were made more convenient by altering the horizontal shelf position and therefore the accessibility (healthier bread is encountered either first or last during visits to the bread section). We hypothesize that the purchase of a certain category of bread is higher when it is encountered first, as it is more visible and prominent than when it is encountered last.

## Methods

### Setting

**Fig 1:**
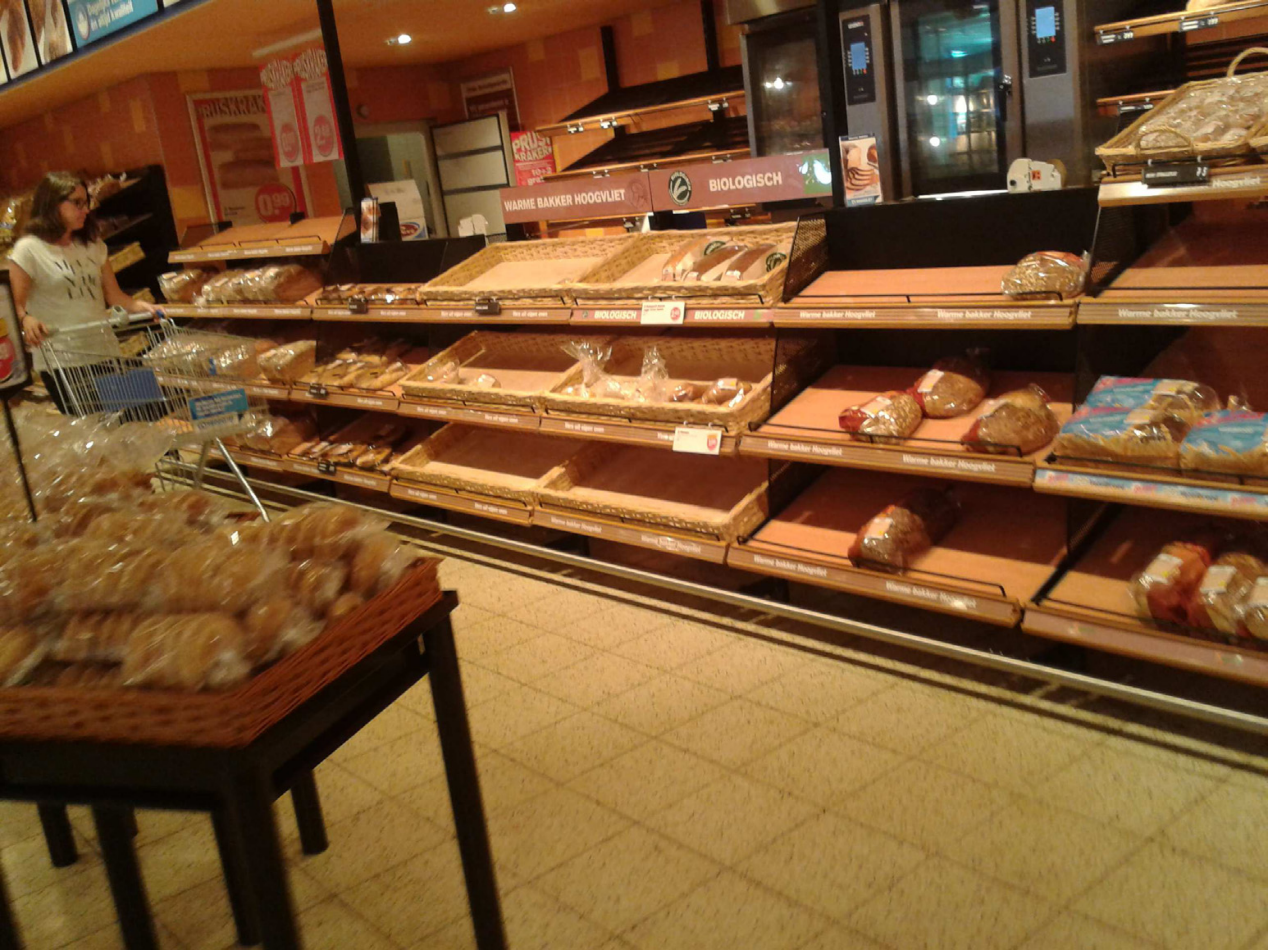
Fresh bread department located near the entrance of the test supermarkets. Shoppers enter the bread aisle from the left and exit from the right to other departments.

Data were based on sales of every type of fresh bread sold in two supermarkets (“supermarket A” and “supermarket B”) in the same chain located in Veenendaal, the Netherlands. Both supermarkets shared a similar store layout. The fresh bread department of both stores was located next to the entrance, and all customers passed this department before they entered the rest of the store. Five clusters of fresh breads were identified, labelled for this study as whole grain (9 types), dark wheat (4), wheat (6), light wheat (4), and white (8) because, in Dutch supermarkets, bread is identified by colour rather than by degree of refinement of the wheat. These bread clusters were organized on shelves on the left side of one aisle that customers entered from the left and exited from the right (see Fig 1). An additional cluster of banquet/bake-off breads (84 types) was situated on the far left and a cluster of specialty bread (26 types) on the far right of the aisle. Shelf height ranged from approximately 30 cm to 150 cm, shelf width was eight and nine metres in supermarket A and B, respectively.

### Experimental design and procedures

**Fig 2:**
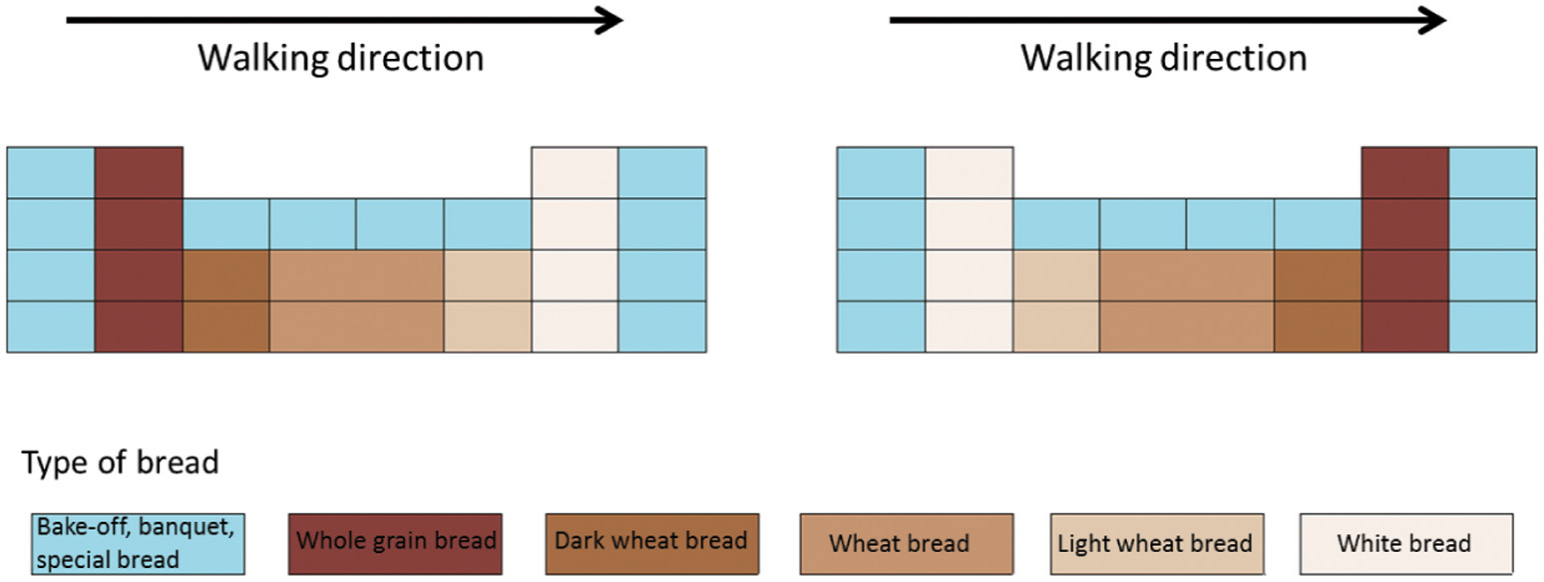
Diagram of bread placement in supermarket.

In the “healthier bread first” condition, whole grain bread was placed at the entrance of the aisle (next to the banquet/bake-offs), followed by dark wheat bread, wheat bread, light wheat bread, and white bread. In the “healthier bread last” condition, the order was reversed, with whole grain bread placed at the exit of the aisle, just before the specialty bread (Fig 2).

The effect of horizontal shelf location was investigated over two periods. During period 1 (week 43–49, 2013), bread was presented in the “healthier bread first” condition in one supermarket (location B) and in the “healthier bread last” condition in the other supermarket (location A). During period 2 (week 5–9, 2014), this order was reversed. Ideally, the second period should have been as long as the first period, but the second period was shortened due to the fact that the original shelf placement needed to be restored in view of upcoming holiday sales. The supermarket staff refilled shelves during the day. Researchers visited both supermarkets daily to verify the bread placements over the shelves.

### Measurement and statistical analysis

The dependent variable was the total number of units of bread sold per day from each of the five bread categories. Since breads were sold as wholes and halves, all sales were recalculated to one whole unit, e.g. one half bread was recorded as 0.5 unit of bread. Day of the week and number were recorded per location, as well as bread promotions. Both supermarkets offered the same bread promotions at the same time, but promotions varied per week in both experimental periods.

The supermarkets differed in the absolute number of bread units sold. To compensate for these differences in absolute sales, and to correct for weekly bread promotions, ratios between the bread sales of both supermarkets were calculated based on average weekly sales during both periods. Thus, for each bread type, the ratio “Sales in Supermarket A”:“Sales in Supermarket B” was calculated for each week. An effect of accessibility on whole grain bread sales should result in relatively more whole grain sales in supermarket B during period 1, where it was more accessible, and relatively fewer sales during period 2 compared to supermarket A. As it was expected that a carry-over effect might occur that affected wheat bread sales, in the analysis wheat bread was divided into three categories based on colour.

## Results

### Bread sales: General

Absolute numbers of sold units of breads were used to verify statistical differences between bread type, day of the week, and supermarkets. Bread sales in supermarket B significantly exceeded bread sales in supermarket A on average by 68% during both periods. Despite differences in absolute number of bread sales, both supermarkets showed similar effects of day of the week and type of bread (Table 1). Bread sales differed systematically with day of the week, with lowest sales on Mondays and Tuesdays and highest sales on Fridays and Saturdays. Repeated measures ANOVA showed significant differences in sales per day of the week for both supermarket A (F (2.52, 108) = 130, *p* < .000, *eta*^2^ = .75) and supermarket B (F (2.15, 92) = 112, *p* < .000, *eta*^2^ = .72).

**Table 1:** Total number of bread units sold per day of the week, supermarket, and test period/condition.

	Period 1		Period 2	
	*Supermarket A*	*Supermarket B*	*Supermarket A*	*Supermarket B*
	*Less healthy bread first*	*Healthier bread first*	*Healthier bread first*	*Less healthy bread first*
**Monday**	88	140	81	135
**Tuesday**	96	122	90	116
**Wednesday**	96	163	92	154
**Thursday**	102	188	89	167
**Friday**	116	239	109	206
**Saturday**	146	229	134	216
**Average**	107	180	99	166

Sales of whole grain bread were significantly higher (32.5% of total bread sales) than sales of dark wheat bread (17.9%), wheat bread (25.0%), light wheat bread (9.7%), and white bread (14.9%) (Table 2).

**Table 2:** Percentage and number of bread units sold per type, supermarket, and test period.

	Less healthy bread first		Healthier bread first		Total sales
	*Supermarket A (period 1)*	*Supermarket B (period 2)*	*Supermarket A (period 2)*	*Supermarket B (period 1)*	
**Whole grain**	34% (178)	31% (251)	34% (166)	33% (289)	32.5% (884)
**Dark wheat**	17% (89)	18% (150)	17% (85)	18% (162)	17.9% (486)
**Wheat**	25% (132)	26% (216)	25% (122)	24% (209)	25.0% (679)
**Light wheat**	9% (47)	10% (85)	8% (38)	11% (93)	9.7% (263)
**White**	16% (83)	14% (117)	16% (75)	15% (128)	14.9% (403)
**Total sales**	100% (529)	100% (819)	100% (486)	100% (881)	100% (2715)

### Bread sales: Effect of accessibility

**Fig 3:**
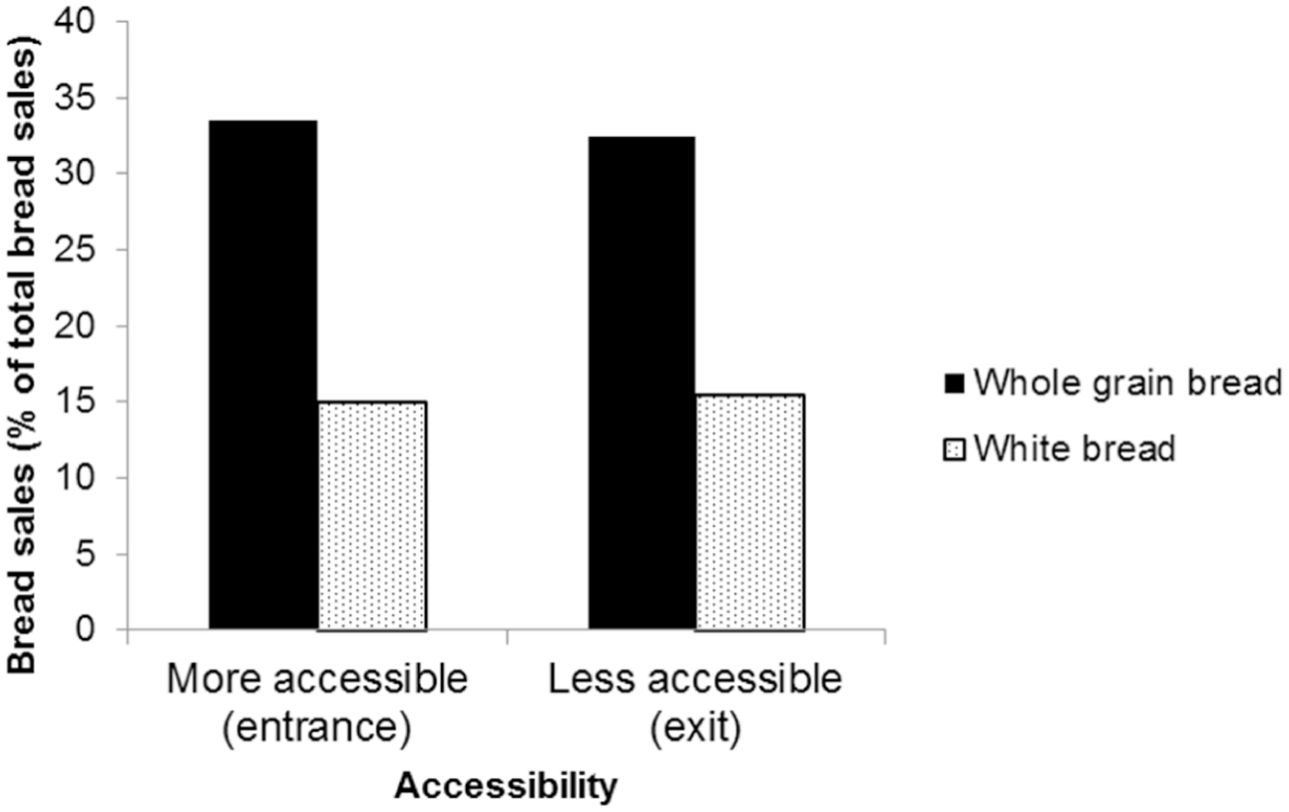
Effect on sales of bread placement.

To investigate an effect of accessibility of whole grain bread across both supermarkets, a 5x2 ANOVA with type of bread (whole grain, dark wheat, wheat, light wheat, white) and condition (“healthier bread first”, “healthier bread last”) as between-subject factors on average week sales ratio was performed. A main effect was found on the average sales ratio between supermarkets for bread type (*F* (4, 55) = 13.0, *p* < .001, *eta*^*2*^ = .487), but not for time period (*F* (1, 55) = 1.90, *p* = .174, *eta*^*2*^ = .033), nor for the interaction between type of bread and time period (*F* (4, 55) = 1.95, *p* = .115, *eta*^*2*^ = .124). Thus, the sales of bread type differed in the two supermarkets. However, no effects of condition, or an interaction between condition and type of bread, were found. Hence, accessibility did not affect sales (Fig 3).

## Discussion

The unplanned character of most in-store consumer decisions coupled with the automatic and habit-driven nature of many consumer decisions offer opportunities to gently direct—or nudge—consumers towards healthier foods. In the present study, the effect of a convenient shelf space location on the sales of whole grain and white bread was examined in two supermarkets by varying their accessibility. In the accessible, healthier bread first condition, whole grain bread was placed in a more convenient position on the left side of the shelves in the bread section (Fig 2). In this condition, customers encountered the healthier bread first. This order was reversed in the less accessible, healthier bread last condition.

Overall, the results showed no effects of our manipulation on sales of the different bread types. That is, sales of whole grain bread were not affected by placement in a more or less convenient location. A number of reasons might explain this lack of effect. First of all, it can always be argued that longer intervention periods and more test supermarkets could have produced different results. However, the intervals used and the low number of test supermarkets did show consistent and significant effects of variables such as the day of the week, specific weeks, and promotions on bread sales (not shown). In view of these significant effects, we believe that a lack of significant convenience effect demonstrates that it is either absent or very small and therefore irrelevant.

Second, consumer bread preferences and purchase habits are strong, and this may lead to a less ‘nudgeable’ product. Nudging requires the right amount of strength to influence decision-making processes. The fact that most consumers buy the same type of bread even when its location in the store changes suggests that consumers have strong bread preferences, at least with regard to white and whole grain bread; a typical consumer of white bread may not select whole grain bread instead just because whole grain bread is encountered first, and vice versa. This effect may be especially strong for white and whole grain bread, which may appeal to very different consumer segments [28–29]. Other types of bread such as whole grain and wheat bread may be more interchangeable for consumers [30]. Consumers often believe that the darker the bread, the healthier it is. In retrospect, varying the accessibility of whole grain and wheat bread could have produced more positive effects in this study.

Bread is also a product that is typically purchased on a regular basis, sometimes even daily. This, plus the fact that bread does not trigger purchasing behaviour like products such as beer or fatty food, means that the purchase of bread is most often habitual and planned [13]. The habitual and planned nature of bread purchases could refer to either the location of specific breads or the bread type itself. If bread purchases are driven by location, switching breads to different locations should lead to a shift in sales from one type of bread to another. The fact that this does not happen in this study suggests that habit refers to the type of bread that is purchased and that customers will look for that preferred bread in the event of relocation. The preferred bread category may be broader than one specific type of bread and may include for example whole grain as well as wheat bread; this could be the subject of further studies.

Finally, the effectiveness of nudges may be very context-specific. A question that arises is whether the convenience and the prominence of the bread were really varied. In other studies, the convenience of product selection is varied for example by placing the products closer to or further away from the shopper [17], or by placing them at the beginning or at the end of a line buffet [22]. In the former case, it takes more effort to select the product that is further away. In the latter case, the assumption is that it is more convenient to select the product at the beginning of the line rather than risk the possibility of encountering no more attractive alternatives in the rest of the line and having to go back (often against the traffic). We had speculated that our supermarket intervention would resemble the line buffet intervention. However, there are some critical differences. Going against the traffic may be much more problematic with a line buffet than in our supermarket situation because of the amount of traffic and the available space to manoeuvre. As Fig 1 clearly illustrates, neither factor is typically really relevant in the test supermarket. Consequently, (perceived) accessibility may have been similar for the breads placed at the entrance and at the exit of the bread department.

In summary, the results of the study demonstrate no effect on sales of placement of bread near the entrance or near the exit of the bread department of supermarkets. Future studies may include other product categories and other types of product placements in supermarkets to explore whether the lack of effect in this study is related to the bread product category and/or to the good accessibility of the products within this category.
